# A Review of Ocular Drug Delivery Platforms and Drugs for Infectious and Noninfectious Uveitis: The Past, Present, and Future

**DOI:** 10.3390/pharmaceutics13081224

**Published:** 2021-08-08

**Authors:** Christopher D. Conrady, Steven Yeh

**Affiliations:** 1 Department of Ophthalmology and Visual Sciences, Truhlsen Eye Center, University of Nebraska Medical Center, Omaha, NE 68105, USA; 2 Department of Ophthalmology and Visual Sciences, Kellogg Eye Center, University of Michigan, Ann Arbor, MI 48105, USA

**Keywords:** drug delivery, eye, uveitis, inflammation

## Abstract

Uveitis refers to a broad group of inflammatory disorders of the eye that often require medical and surgical management to improve or stabilize vision and prevent vision-threatening pathological changes to the eye. Drug delivery to the eye to combat inflammation and subsequent complications from uveitic conditions is complex as there are multiple barriers to absorption limiting availability of the needed drug in the affected tissues. As such, there has been substantial interest in developing new drugs and drug delivery platforms to help reduce intraocular inflammation and its complications. In this review, we discuss the challenges of drug delivery, novel technologies recently approved for uveitis patient care and promising drug delivery platforms for uveitis and sequelae of ocular inflammation.

## 1. Introduction

**Figure 1:**
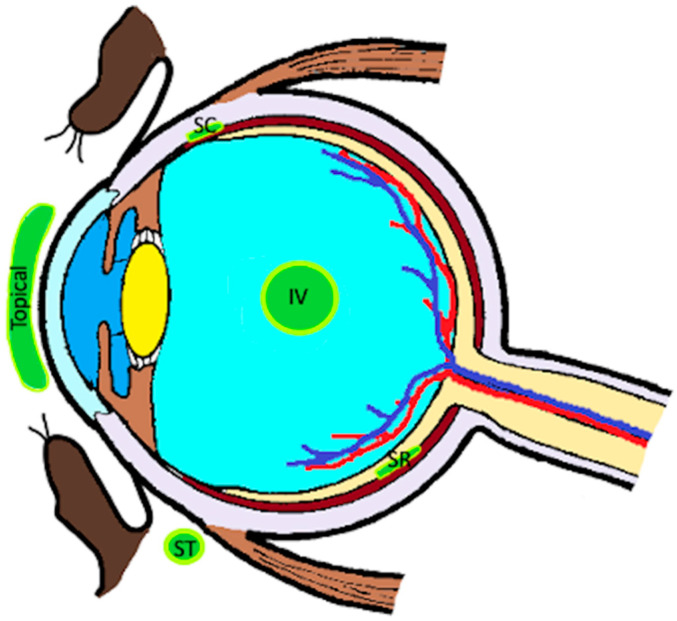
Routes of local drug delivery. IV, intravitreal; SC, suprachoroidal; SR, subretinal; ST, subtenon’s.

**Figure 2:**
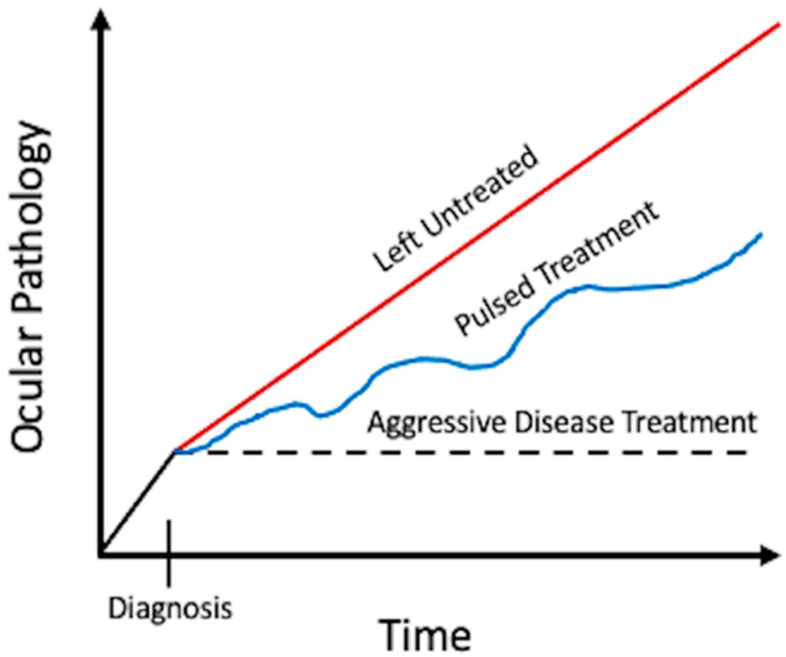
Our hypothesized rate of pathological changes in a uveitic eye with or without treatment. Red line, no treatment; blue line, periodic steroid pulses (either locally or systemically), black dotted line, adequately treated uveitis.

Adequate drug bioavailability in and around the eye is difficult to achieve due to multiple local barriers, and this is especially important in inflammatory diseases of the eye. In addition to adequate inflammatory and pathogen control, ocular hypertension, cystoid macular edema, and corneal edema are common complications of uveitis that may require medical therapy, and in some cases, surgery (Table 1) [1]. To further complicate matters, inflammation or complications related to inflammation may affect structures in or around the eye simultaneously, and in some cases, sequentially with disease recurrence [2]. Based off of emerging uveitic literature, pulsed therapy or undertreated disease, we hypothesize, leads to increasingly recalcitrant and sometimes irreversible ocular pathology over time (Figure 1; Figure 2) [3]. Thus, the necessary aggressive medical treatment of uveitic disorders, both infectious and noninfectious, can be quite complex and require multiple methods of drug delivery to quell disease. As more data emerges, it is clear that early and aggressive intraocular inflammatory and macular edema control is crucial to maintain good visual acuities in patients with uveitis long term [3]. Consequently, expanding drug delivery choices and alternatives, and improving bioavailability of available medications, while balancing safety measures, is important in treating uveitis and will be discussed in more detail in the following review with an emphasis on emerging drugs and/or techniques.

## 2. Overview of Drug Delivery Systems

There a several routes that drugs can be delivered to the eye. They include systemic (i.e., oral or parenteral routes which may include intravenous or subcutaneous routes), topical, periocular (i.e., subconjunctival, subtenon’s, suprachoroidal), or intravitreal administration, with the most common route being topical administration of eye drops (Table 2, Figure 2). Most non-systemic routes of administration require inoculation of the drug directly into the targeted ocular space or absorption across the cornea, conjunctiva, and sclera [4,5].

There are inherent barriers to drug delivery within and around the eye. For example, only approximately 5% of a single eye drop is absorbed and reaches intraocular tissues [6]. This low drug absorption is due to rapid removal from the ocular surface through the lacrimal drainage system and systemic absorption in the conjunctival sac as well as physical barriers to absorption, mainly the corneal epithelium [6]. To complicate matters, targeting intraocular tissues with systemic administration of drugs is limited by the blood–retinal and blood–aqueous barriers [7]. In eyes with inflammation, the situation becomes even more complex as the blood–retinal and blood–aqueous barriers are likely compromised and may allow drugs into the eye that otherwise would have not entered the eye. In uveitis patients poor drop adherence can further complicate matters as seen in glaucoma patients [8,9]. However, limited data exists on the impact of these barrier changes with inflammation and drug delivery to the eye.

Furthermore, the pharmacokinetics of many ocular therapeutics in patients including their ocular penetration, pharmacodynamics, and durability is incompletely understood. The few published studies on ocular pharmacokinetics are typically single drug measurements in humans or repetitive drug measurements in rabbits and have been reviewed elsewhere [4,5,10,11]. In the following review, we will discuss current therapies and systems or those in clinical trials that may be available in the future.

## 3. Systemic Medications

### 3.1. Systemic Immune Modulators and Their Use Locally

Systemic steroids have been the mainstay of treatment for bilateral non-infectious uveitis given their efficacy, particularly for acute, vision-threatening disease. However, oral or intravenous steroids can lead to significant systemic side effects even at low doses including loss of glycemic control, significant weight gain, hypertension, psychoses, increased risk of infection, osteoporosis, poor wound healing, and gastrointestinal disturbances including perforation [12]. Similarly, administration of oral non-steroidal anti-inflammatory drugs (NSAIDs) have been utilized to prevent and treat active uveitis as safer alternatives to systemic steroids but can also have significant side effects (most notably gastrointestinal bleeding and/or perforation) [13,14]. Consequently, the search for more specific steroid-sparing agents with fewer side effects and that are more efficacious, longer acting local therapies has gained significant interest to quell intraocular inflammation.

The advent of immune modulatory therapy (IMT) as a safer long-term alternative to steroids was first pioneered in the 1970s with US Food and Drug Administration approval of the use of low dose methotrexate to treat psoriasis. Since that time, there has been a rapid expansion in IMT medications including the development of oral (cyclosporine, mycophenolate), subcutaneous (adalimumab), and intravenous biologics (infliximab, more targeted therapy) to target various inflammatory pathways or specific cytokines throughout the body. Biologics (adalimumab, infliximab, etc.) have also been used to treat all types of noninfectious uveitis with varying success [15,16,17]. These drugs have only added to the armament of well-tolerated and previously well-studied anti-metabolites to treat non-infectious uveitis (Table 3) [18,19]. These drugs have been shown to reduce inflammation, improve macular edema, and in some cases, lead to disease remission. As such, these systemic medications remain an essential tool for any uveitis specialist when utilized appropriately [1,19,20,21]. There is an extensive list of systemic emerging therapeutic agents that have been discussed in detail elsewhere and many more biologics that are currently under exploration [22].

Several IMT drugs have been used locally in preclinical models, and others even clinically, with varying rates of success to treat macular edema and/or intraocular inflammation. The first such drug, methotrexate, appeared to have some benefit in a retrospective case series in treating macular edema, and in another small series, allowed a reduction in systemic IMT when injected intravitreally [31,32]. The drug’s use in treating macular edema is currently under further investigation as part of the Macular Edema Ranibizumab versus Intravitreal Anti-inflammatory Therapy (MERIT) multicenter, randomized controlled trial with results due in the near future. Additionally, intravitreal injections of numerous anti-tumor necrosis factor (TNF) agents have been used in several small, non-randomized studies with mixed results [33]. Liposomal preparations of infliximab, a chimeric anti-TNF antibody, are being developed to prolong the medication’s duration within the vitreous and has shown some success in mice [34]. Unfortunately, there is little-to-no safety data available from these studies and the Fc region of the anti-TNF antibody is known to be immunogenic with systemic administration raising concerns with intraocular injection [35]. Due to these safety concerns with anti-TNF agents administered intravitreally, a group has removed the Fc portion of infliximab, which has shown to decrease immunogenicity but remains a potent, intravitreal biologic in mice [36].

Systemic administration of sirolimus, a drug used to prevent organ transplant rejection, has been studied in preclinical uveitis models and in patients with some success but requires monitoring for systemic toxicity [37,38]. A modified formulation of the drug for intravitreal injection has been shown to reduce vitreous haze and increase the likelihood to successfully taper systemic corticosteroids with no reported ocular toxicity in posterior uveitis in masked trials [29,39]. Subsequent dose de-escalation studies have shown that 440 µg of the drug appear to be most efficacious with minimal associated-to-no toxicity [29].

In rabbits with severe uveitis following injection of *Mycobacterium tuberculosis* antigen, a prolonged infusion (1 h or more) of sub-Tenon’s cyclophosphamide resulted in high concentrations of the drug within the vitreous and retina and a significant improvement in inflammation compared to controls [40]. There was minimal systemic absorption found [40]. While this proof-of-concept study was interesting, these results seem impractical to replicate clinically currently due to the need of an extended sub-Tenon’s infusion through a catheter unless another, more rapid delivery platform can be developed. However, local administration of cyclophosphamide likely limits its systemic toxicity and may expand its use [41]. Thus, the local and systemic use of IMT agents is rapidly expanding and will likely to continue as more agents are developed, toxicities better studied, and clinical trials are designed to evaluate their efficacy in non-infectious uveitis. It remains to be seen whether local therapy can induce remission of non-infectious uveitis like their systemic counterparts.

### 3.2. Systemic Antibiotics

The largest, randomized, controlled trial on post-cataract surgery endophthalmitis, Endophthalmitis Vitrectomy Study (EVS) found no additional benefit of intravenous antibiotics in visual outcomes compared to intravitreal therapy alone [23]. Furthermore, most available systemic antibiotics have either not been studied or fail to reach concentrations able to inhibit intraocular bacterial growth. A recent review by Brockhaus et al. compiled all available studies on intravitreal penetration of systemic antibiotics but are outside the scope of this review [7]. Consequently, systemic administration of antibiotics for intraocular infections is controversial, while there is a clear role for intravitreal injection of the medications [23].

**Figure 3:**
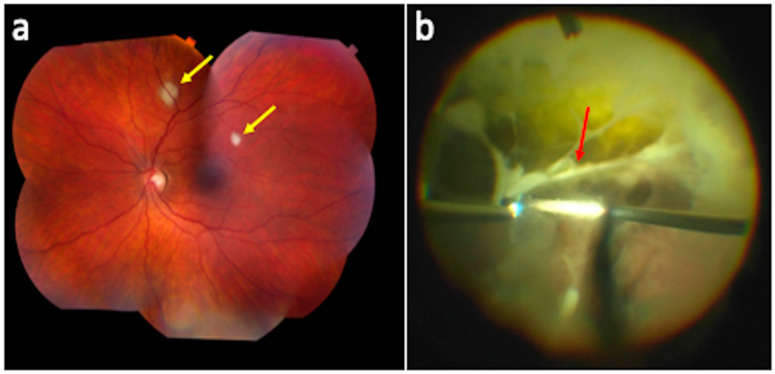
Intraocular fungal complications. A patient with candida chorioretinitis (**a**, yellow arrows) versus a patient with candida chorioretinitis with a “string of pearls” (**b**, red arrow).

On the other hand, the standard treatment of care for endogenous fungal chorioretinitis is systemic antifungals and is usually sufficient in cases that lack sight-threatening lesions or vitreous involvement [42]. Additionally, peripheral toxoplasmosis chorioretinitis is routinely managed with systemic medication alone. Thus, eradication of the pathogen can be successfully achieved with systemic therapy through either oral or intravenous routes alone. The EVS had some significant limitations, most notably that it utilized intravenous ceftazidime, which has poor activity against the most common Gram (+) organisms, and amikacin, which has very poor intraocular penetration [7,23,42]. The emergence of fluoroquinolones, specifically moxifloxacin, which has shown to concentrate within the vitreous at concentrations required to inhibit bacterial growth, has led some to question recommendations from the EVS that systemic antibiotics are of no additional benefit in the battle to sterilize the aqueous and vitreous of bacterial infection [7,42]. Until a large, randomized study using newer, better intraocular penetrating systemic antibiotics is performed, the use of adjuvant oral or intravenous antibiotics will remain controversial. We hypothesize by extrapolating from the EVS and much like the treatment of endogenous endophthalmitis or sight-threatening fungal chorioretinitis (Figure 3), where both intravitreal and systemic antibiotics are utilized, cases of post-procedural and -traumatic endophthalmitis may benefit from the addition of systemic antimicrobials that rapidly equilibrate to high concentrations within the vitreous after intravitreal antimicrobials have been administered [43]. However, a randomized, controlled trial is needed with newer generation systemic antibiotics to evaluate their benefit in endophthalmitis of any cause.

To compound matters, there are unfortunately no systemic antibiotics designed specifically to penetrate/treat infections of the eye. Additionally, there has been a paucity of resources dedicated to antibiotic development despite the emergence of rare and resistant pathogens isolated from the eye and elsewhere [44,45,46]. Hopefully in the future, more systemic antibiotics with better bioavailability within ocular tissues will become readily available.

## 4. Local Therapy

There are several established routes of local ocular delivery and they include topical eye drops, subconjunctival injections, sub-Tenon’s injections, intravitreal injections, surgical implants, and the newest route, suprachoroidal injections. Each route has unique benefits and risks with differential risk depending on the drug and route of administration. For example, rare but potentially vision-threatening risk attributable to intraocular procedures such as surgical implants or drugs requiring injection into the eye, include endophthalmitis and retinal detachment. More commonly, patients who receive corticosteroids given topically or local injection may be at increased risk of ocular hypertension and cataract.

### 4.1. Topical Medications

Topical drops are the mainstay of any ophthalmology practice, particularly in a uveitis practice. Due to ocular complications associated with uveitis, providers must be well versed in topical steroid and anti-hypertensives. Particularly important to uveitis patients due to the diseases’ long duration, poor drop adherence is well documented within glaucoma patients and special training may be required to improve compliance [8,9]. Uveitis patients are similar to their glaucoma counterparts. Additionally, frequent topical steroid use has been associated with cataract development [47]. Thus, topical drops, like any other medication, should be used with caution and with proper patient education. To focus this review, we will avoid reviewing all topical eye drops (i.e., steroids, anti-hypertensives, and antibiotics) as they have been routinely used in ophthalmology practices for many years and the emerging derivatives will be discussed later in further detail. It is worth mentioning, though, that drugs such as difluprednate are stronger than 1.0% prednisolone acetate and the even weaker 0.1% fluorometholone [48,49].

### 4.2. Sub-Tenon’s Injections

Sub-Tenon’s injections of triamcinolone (most commonly, 40 mg is given through a supero- or inferotemporal injection (transseptal) through a cannula or needle) have been effectively used to treat intraocular inflammation and uveitic macular edema for some time with maximal effect approximately one month after injection [50]. The sub-Tenon’s route of administration is likely safer than intravitreal injections as the procedure (if no complications occur such as globe perforation) does not require entering the eye eliminating needle-associated risks (endophthalmitis, retinal tears, etc.) [51]. Worsening cataracts, elevation in intraocular pressure, and lower efficacy in treating uveitic macular edema compared to intravitreal steroids in a large randomized, controlled trial, has reduced this route’s use with other alternatives available [28,50]. However, this route of delivery remains an important tool in any uveitis expert’s hands, especially in those cases where intravitreal injections may not be possible or require extraordinary efforts (pediatric uveitis). A small retrospective study suggested subconjunctival triamcinolone was equally efficacious treating uveitic macular edema as the sub-Tenon’s route; however, true, head-to-head testing has not been done [52]. As with any periocular or intraocular steroid injection, ocular hypertension and worsening cataracts are possible with subconjunctival or sub-Tenon’s triamcinolone [53].

### 4.3. Suprachoroidal Injections

Suprachoroidal injections are a relatively new method of drug delivery to the eye and are being tested in genetic disorders, macular degeneration, diabetic macular edema, ocular oncology, and non-infectious uveitis [54,55]. Initial preclinical studies have shown that injection of fluorescein dye into the suprachoroidal space resulted in higher concentrations of the dye in the choroid and retina than either intravitreal or subconjunctival routes [56]. Furthermore, concentrations of triamcinolone remain fairly localized to the retina, choroid, and sclera following suprachoroidal injection with minimal exposure of other steroid-sensitive ocular structures (i.e., lens and cataract formation) [57]. The drug also appears to remain fairly localized to the posterior segment (choroid, retina, and vitreous) as concentrations of the drug remain very low in the anterior segment and systemically following injection [57,58].

This preclinical data has also been seen clinically. In one of the original pilot studies, all seven patients with non-infectious uveitis had improvement in macular edema, but equally important, had no documented episodes of ocular hypertension in the 26 weeks following suprachoroidal injection of triamcinolone [59]. In follow up phase I/II clinical trials, suprachoroidal injections of triamcinolone were well tolerated with minimal side effects and no documented adverse events related to the injection [59,60]. Much like the preclinical studies, minimal systemic absorption was found [60]. In the most recent phase III trials, the suprachoroidal injection resulted in a significant reduction in macular edema and visual improvement compared to sham injections [30,61]. The injection has been marketed to target and treat macular edema; however, there is hope that this new method could be another approach to treating posterior segment inflammation but with a lower side effect profile than other steroid-based therapies.

### 4.4. Intravitreal Injections

Intravitreal steroids are frequently utilized medications for intravitreal inflammation and uveitic macular edema, while reducing the systemic side effects of oral steroids. Local steroids alone or in combination with other local or systemic anti-inflammatory medications can be used to treat noninfectious uveitis. Intravitreal triamcinolone is quite effective treating macular edema for an average of 5 weeks with repeated administration possible [62]. Much like other steroids, ocular hypertension and cataract development are not infrequent complications that may require future surgery [62]. Additionally, intravitreal triamcinolone can make distinguishing endophthalmitis from vision loss related to the medication itself difficult clinically as patients may not have eye pain or even conjunctival injection but have a hypopyon [63,64].

There are several steroid-containing intravitreal implants on the market with varying lengths of duration and efficacy. The first FDA-approved injectable implant was the sustained release 0.7 mg dexamethasone (Ozurdex) pellet that has been shown to be safe and effective for uveitic macular edema, improving best corrected visual acuity and reducing vitreous inflammation of patients with non-infectious uveitis. Much like intravitreal triamcinolone, repeated injections even in children and in eyes that have been vitrectomized is possible and effective [65,66,67,68,69]. Like many other steroid formulations, intraocular pressure elevation and cataract development can occur [70]. The larger gauge needle on the ozurdex injector can also result in significant wound leaks and even hypotony most commonly in post-vitrectomized eyes [71]. Despite these risks, in the largest randomized trial of its kind, the POINT trial found that intravitreal triamcinolone or intravitreal dexamethasone implants outperformed periocular triamcinolone injections to treat uveitic macular edema [28]. This is balanced by greater odds of elevated intraocular pressure associated with intravitreal corticosteroid compared to periocular administration.

There are two available intravitreal fluocinolone acetonide inserts (YUTIQ 01.8, Illuvien 0.19 mg). They are similar to the Retisert implant (discussed later) in that they are long-acting (approximately 3 years) local steroids; however, the inserts can be injected in clinic and release lower doses of steroids than the Retisert that must be surgically implanted [72,73,74]. These injectables have been shown to reduce rates of noninfectious uveitis recurrence, improve uveitic macular edema, and in birdshot chorioretinitis, reduce vascular leakage [26,75,76]. The lower intraocular concentrations of fluocinolone have been associated with lower rates of ocular hypertension than with the Retisert implant [72]. While we do not have extensive experience with either medication, there has been some concern that the concentrations of medication released is not high enough to control uveitic inflammation alone and should be used to supplement systemic IMT. Additionally, there can be complications associated with the medications requiring medical, and in more severe cases, surgical interventions [77].

## 5. Surgical Implants

There have been several intraocular implants that require surgical implantation developed and used clinically to treat viruses and inflammation. The slow-release ganciclovir intraocular implant (Vitrasert, Bausch and Lomb) for cytomegalovirus (CMV) retinitis showed superior efficacy in local control and delay to disease recurrence when compared to intravenous ganciclovir in CMV retinitis associated acquired immunodeficiency syndrome. However, the implant delivered medication for a modest duration (~ 5–8 months) and complications were observed in relationship to the implant and/or implantation procedure. Patients also continued to require anti-CMV treatment to avoid contralateral eye disease and to prevent morbidity and mortality associated with CMV viremia [25,78,79]. The advent and widespread use of antiretroviral therapy given HIV, reduced usage of the ganciclovir implant and other factors such as orally administered valganciclovir led to the discontinuation of production of the ganciclovir implant, but was an important step in drug development and longer acting, implantable drug delivery systems [24].

Similarly, the non-biodegradable 0.59 mg fluocinolone acetonide implant (RetisertTM, Bausch and Lomb) may be employed in the treatment of non-infectious uveitis. The implant is quite effective clinically and has been shown to reduce uveitic recurrences and improve visual acuities while reducing the need for additional adjunctive anti-inflammatory medications [27]. However, a high rate of cataract requiring surgery, 80%, and development of glaucoma requiring filtration surgery in approximately 26% of individuals receiving the fluocinolone acetonide implant are long-term considerations that warrant monitoring. The high cost and insurance coverage considerations may also be prohibitive for some patients [80,81,82]. In a small, comparative case series, the implant performed as well as the dexamethasone-containing injectable, (Ozurdex), but had greater rates of cataract development and intraocular pressure spikes [81]. In a subsequent study, 74.8% of patients required topical antihypertensives, while 36.6% of patients would require incisional glaucoma surgery by 36 months following surgical implantation of the device [83]. However, the fluocinolone implant has a much longer duration of activity than the dexamethasone injectable (3 years versus 2–3 months) making it a viable long term option in those patients that cannot tolerate systemic steroids and IMT but uveitis remains active [19,27,84]. Additional implants can be inserted extending the duration of treatment available with this sustained release drug delivery implant [84]. Due to the wound size required for implantation, associated scleral thinning and rare reports of scleral melt, scleral integrity should be monitored, particularly in patients undergoing reimplantation [85,86].

## 6. The Future

There are several drug delivery platforms in the pipeline (Table 4) for other ocular conditions or are being developed in research labs that may be of some benefit to the uveitis community in the future. They include topical nanotechnology, drug reservoirs, immunotherapies, gene/plasmid therapy, and drug-eluting contact lenses.

While not new to the field of medicine but an important treatment in persistent CMV infections, adoptive immunotherapy with CMV-specific cytotoxic T lymphocytes has shown promise in cases of persistent or progressive retinitis or viral resistance [87]. While these clinical results were retrospective in nature, the approach has been used to successfully treat CMV resistance in other organs resulting in improved survival and provides protection from CMV-related death [88,89]. Recent research has taken this idea a step further to test whether adoptive transfer of drug treated T cells or clonally expanded T regulatory cells could impact intraocular inflammation in non-infectious uveitis. In early mouse studies, T cells treated with immune regulatory agents such as teriflunomide or activated T regulatory cells were administered intravenously or intravitreally and inhibited the development of non-infectious uveitis [90,91,92]. Conversely, locally or systemically depleting mice of T regulatory cells worsens and/or delays resolution of uveitis [93,94]. While these experimental results are promising, there have been well documented cases of patients losing vision following intravitreal injection of autologous stem cells for various disorders [95]. Thus, systemic and ocular safety must be fully evaluated before these therapies become available, especially if intraocularly injected.

Nanotechnology is a promising field of study for ocular drug delivery including uveitis by using nanoparticles as carriers to improve delivery of drugs of interest to the necessary end organ. Polymeric nanoparticles have been shown in rabbits to prolong the duration of the loaded drug resulting in higher and longer anti-inflammatory activity in the eye than the drug alone [96]. Topical polymeric nanomicellar formulations of voclosporin have been shown to penetrate the cornea and lead to high aqueous concentrations of the drug and improvement of dry eye in preclinical models [97]. Eye drops composed of microparticles containing dexamethasone-cyclodextrin have been shown to be well tolerated in a small pilot study of patients with diabetic macular edema [98]. Optimization with thiolation, amino acid modifications and PEGylation has improved corneal penetration of larger nanoparticles expanding the list of drugs that could be delivered to the eye with these aforementioned nanoparticles [99,100]. In other experimental data more specific to uveitis, topical polymeric nanoparticles loaded with triamcinolone or antioxidant enzymes such as superoxide dismutase have been shown to reduce clinical signs of uveitis in rabbits [101,102]. Subconjunctival, controlled-release, carboxyl-poly lactic-co-glycolic acid, steroid-containing nanoparticles are also being optimized and have been shown to significantly reduce inflammation in rat models of uveitis [103]. Even systemically administered polyester nanoparticles loaded with curcumin, a potent anti-inflammatory extract, significantly reduced ocular inflammation in a lens-induced uveitis model [104,105]. Unfortunately, many of these aforementioned formulations of nanoparticles are in very preliminary stages of investigation and nanoparticle distribution and elimination in the eye are not well understood at this time [106].

Maybe the furthest along in development and currently in phase III clinical trials after promising results in phase II trials, the port delivery system is a novel, permanent refillable surgical implant that is filled with ranibizumab for neovascular age-related macular degeneration [107]. Similarly, a porous subconjunctival drug delivery system of microspheres has been shown to deliver bevacizumab and anti-inflammatory antibodies to the cornea and retina in rabbits [108,109,110]. As more anti-inflammatory agents are being developed/studied as intravitreal agents to combat intraocular inflammation and macular edema in uveitis patients as discussed earlier, it remains to be seen, if, and how these novel delivery systems can be adapted to treating uveitis. The expanding local therapeutic options that could be potentially injected into these reservoir systems for longer, local treatment than with a single intravitreal administration could become quite extensive and specific.

Much like with other diseases of the eye, groups are attempting to use gene therapy to promote non-infectious uveitis quiescence. A non-viral plasmid encoding soluble TNF receptors that can be electrotransfected into the cells of the ciliary body has shown promise in multiple mouse and rat models [111,112,113]. These plasmids have been shown to significantly improve histopathological inflammatory changes and clinical global uveitis scores, a scoring system used to evaluate severity of uveitis in mice and rats [114]. Patients are currently being recruited for phase I/II dose escalation studies of this electrotransfected plasmid. The safety, efficacy, immunogenicity, and long-term stability of these plasmids is still not well known, and are ongoing areas of investigation of even currently approved ocular gene therapies [115,116]. Theoretically, this same delivery system could be used to deliver other, important anti-inflammatory decoy receptors, or even anti-inflammatory chemokines/cytokines to the eye thereby promoting a local, long-lasting anti-inflammatory milieu and non-infectious uveitis quiescence. Could these plasmids be designed to produce neutralizing antibodies for persistent infections (i.e., herpes viruses, toxoplasmosis, etc.) keeping the pathogen under local immune control as well?

Lastly, attempts are being made to produce a contact lens that could deliver medication to the eye effectively bypassing the tear film and its rapid removal from the ocular surface allowing intraocular concentrations of the drug to build over time [117]. In rabbits, the drug-eluting contact lens leads to retinal concentrations of dexamethasone 200 times higher than hourly drops of the medication and inhibition of retinal vascular leakage [117]. There are obvious corneal health concerns as patients that wear contact lenses are at higher risk of developing corneal ulcers and now their ocular surface is being bathed in steroids with the medicated contact lens [118]. Therefore, it remains to be seen, if, and how these lenses are used and whether they put patients at higher risk of developing ocular surface infections with their prolonged use. It may mean that the patient may need to be placed on preventative topical antibiotics or that an antibiotic be added to the contact lens formulations. While development of these lenses is ongoing, their clinical use and indication remain unclear at the present time.

## 7. Conclusions

The delivery of medication requires attention to the challenges of targeted mechanisms of action, barriers to drug delivery, and side effect profile. Uveitis is particularly difficult to treat as prolonged inflammation in the eye results in sequelae that may be difficult to reverse. This is balanced by the risk of local side effects with prolonged or repeated corticosteroid therapies currently available. With more sensitive and specific diagnostic tools for both noninfectious and infectious uveitides becoming available to identify pathways and pathogens not previously known to cause uveitis, targeted therapy will likely become more commonplace [44,45,119,120]. As such, the expansion of targeted corticosteroid delivery and increasing array of non-steroidal treatments, administered both locally and systemically will likely expand our treatment armamentarium. Ultimately, improved understanding of the pathogenesis of specific disease states will facilitate more targeted therapies to avoid undesirable inflammatory complications to improve vision and quality-of-life in patients with sight-threatening inflammatory conditions.

## Figures and Tables

**Table 1:** Uveitic ocular complications requiring medical and/or surgical treatment.

**Media Opacities**
Cataract
Vitreous opacification
Vitreous hemorrhage
Band keratopathy
**Structural Complications**
Cystoid macular edema
Retinal detachment
Epiretinal membrane
Cyclitic membranes
Pars plana snowbanks
Retinal neovascularization
Retinal nonperfusion
Chorioretinal scarring
Posterior syncechiae
Secondary glaucoma
Rubeosis
Papillitis
Papilledema
Hypotony
Phthisis

**Table 2:** Most commonly employed uveitis medications. IMT, immune modulatory therapy; NSAID, nonsteroidal anti-inflammatory drug; subconj, subconjunctival.

Drug	Systemic	Topical	Sub-Tenon’s Subconj	Suprachoroidal	Intravitreal	Surgically Implanted
**Steroid**	Prednisone	Dexamethasone	Triamcinolone	Triamcinolone	Dexamethasone	Retisert
		Difluprednate	Dexamethasone		Triamcinolone	
		Fluormetholone			Ozurdex	
		Loteprednol			Yutiq	
		Prednisolone			Illuvien	
**Steroid** **Alternative**	NSAIDs	NSAIDs				
**IMT**	Adalimumab				Sirolimus	
	Azathioprine				Methotrexate	
	Chlorambucil					
	Cyclophosphamide					
	Cyclosporin					
	Infliximab					
	Methotrexate					
	Mycophenolate					
	Rituximab					
	Sirolimus					
	Tocilizumab					
**Antibacterial**	Daptomycin		Vancomuycin		Amikacin	
	Linezolid		Ceftazidime		Ceftazidime	
	Meroepenem		Cefazolin		Vancomycin	
	Moxifloxacin		Gentamycin			
**Antifungal**	Fluconazole				Amphotericin B	
	Micafungin				Voriconazole	
	Voriconazole					
	5-flucytosine					
**Antiviral**	Acyclovir				Cidofovir	Vitrasert
	Valganciclovir				Foscarnet	
	Valacyclovir				Ganciclovir	

**Table 3:** Major randomized, controlled uveitis drug studies. CMV, cytomegalovirus; IM, intramuscular; IV, intravitreal; IVT intravitreal triescence; ME, macular edema; PO, *per os*.

Infectious	Findings
EVS, 1995 [23]	No additional visual acuity benefit with or without systemic antibiotics; for patients with hand motions vision or better, no difference in final visual acuity with immediate vitrectomy vs. tap and inject of antibiotics
Martin et al., 2002 [24]	PO valganciclovir as efficacious as intravenous ganciclovir for CMV retinitis induction therapy
*Musch et al., 1997* [25]	Ganciclovir implant is more effective than intravenous ganciclovir for CMV retinitis, but patients treated with ganciclovir implant alone were at risk for CMV complications outside of the treated eye
**Non-infectious**	
FAST (Rathinam et al., 2019) [18]	PO Mycophenolate is not superior to PO methotrexate in controlling inflammation in adult uveitis. For posterior and panuveitis, methotrexate was associated with greater treatment success than mycophenolate
*Jaffe et al., 2020* [26]	Injectable fluocinolone insert reduces uveitic recurrences
*Jaffe et al., 2016* [17]	Adalimumab reduces risk of uveitic flare and lower risk of visual impairment when compared to placebo
*Jaffe et al., 2006* [27]	Surgical fluocinolone acetonide intravitreal implant reduced uveitic recurrences, but were associated with increased risk of elevated intraocular pressure and cataract development
MERIT	Evaluating treatment of ranibizumab, dexamethasone implant and methotrexate for ME, NIH-funded study ongoing
MUST (Tomkins-Netzer et al., 2021) [3]	IMT or fluocinolone implant improved uveitic ME. Systemic IMT and the surgical fluocinolone acetonide implant demonstrated mean visual acuity gains over 24 months, with neither group clearly superior. Systemic IMT was well-tolerated.
POINT (Thorne et al., 2019) [28]	IVT or IV dexamethasone are superior to periocular steroids for ME with modest increases in IOP in the intravitreal treatment groups
SAKURA (Merrill et al., 2020) [29]	IV sirolimus improves ocular inflammation with preservation of visual acuity
*Yeh et al., 2020* [30]	Suprachoroidal injection of steroids is safe and efficacious for ME

**Table 4:** Therapies on the horizon. DE, drug-eluting; TNF, tissue necrosis factor; T reg, T regulatory cells.

Drugs	Systemic	Topical	Subconjunctival	Suprachoroidal	Intravitreal	Surgically Implanted
**The Future**	Adoptive immunotherapy	Nanoparticles	Nanoparticles	Plasmids/vectors	T reg cell expansion	Port delivery system
	T reg cell expansion	DE contact lens			Anti-TNF agents	Plasmids/vectors
	Nanoparticles					
